# Off-Label Use of COVID-19 Vaccines from Ethical Issues to Medico-Legal Aspects: An Italian Perspective

**DOI:** 10.3390/vaccines9050423

**Published:** 2021-04-23

**Authors:** Davide Ferorelli, Lorenzo Spagnolo, Maricla Marrone, Serena Corradi, Maria Silvestre, Federica Misceo, Francesco Paolo Bianchi, Pasquale Stefanizzi, Biagio Solarino, Alessandro Dell’Erba, Silvio Tafuri

**Affiliations:** 1 Section of Legal Medicine, Interdisciplinary Department of Medicine, University of Bari, 70121 Bari, Italy; davideferorelli@gmail.com (D.F.); mariclamarrone@hotmail.it (M.M.); serenacorradi10@gmail.com (S.C.); maria-silve@libero.it (M.S.); misceo.federica@gmail.com (F.M.); biagio.solarino@uniba.it (B.S.); alessandro.dellerba@uniba.it (A.D.); 2 Department of Biomedical Sciences and Human Oncology, Aldo Moro University of Bari, 70121 Bari, Italy; frapabi@gmail.com (F.P.B.); pasquale.stefanizzi@uniba.it (P.S.); silvio.tafuri@uniba.it (S.T.)

**Keywords:** COVID-19, SARS-CoV-2, off-label use, vaccine, ethics, forensic sciences, public health, vaccine strategies, safety of vaccines

## Abstract

During the COVID-19 outbreak, the lack of official recommendations on the treatment has led healthcare workers to use multiple drugs not specifically tested and approved for the new insidious disease. After the availability of the first COVID-19 vaccines (Comirnaty Pfizer-BioNTech and Moderna COVID19 vaccine), an authorization was issued by national and international Drug Regulatory Agencies in order to speed up their introduction on the market and their administration on a large scale. Despite the authorization, the off-label use of these vaccines may still be possible especially to answer specific concerns as the lack of vaccine doses, the delay in the delivery of planned doses or the pressure from public opinion and political influence also in relation to the evolution of the pandemic. This paper aims to assess the possible off-label use of COVID-19 vaccines and the ethical and medico-legal implications of this eventuality. The scope of this paper is to point out the possible consequences of off-label use of COVID-19 vaccines and possible mitigation and preventive measures to be taken by healthcare workers involved in vaccination procedures.

## 1. Introduction

The off-label use of medicines and vaccines does not have a unique definition and different interpretations of the subject are indeed provided by national and international Drug Regulatory Agencies as the Food and Drug Administration (FDA), the European Medicines Agency (EMA), and the Italian Medicines Agency (AIFA) [1,2,3,4,5] (Table 1).

However, considering these interpretations as a whole, a comprehensive definition could be derived. Indeed “off-label use” could be referred to as the prescription of an approved drug for an indication, posology, age/population group or route/schedule of administration different than that included in the “label”. The term “label” corresponds to the summary of product characteristics (SmPC) in Europe and the FDA Fact Sheets in the US and refers to the properties and the officially approved conditions of use of a medicine. Therefore the “label” provides the basis of information for healthcare professionals on how to use the medicine safely and effectively.

In Italy, free access to drug therapy for different indications than the approved is possible since 1998 according to Law 94/98 art.3, paragraph 2 (former Law Di Bella) and thus nowadays off-label use is a common medical practice in Italy as in other countries [6]. Indeed, in 1985, Strom et al. pointed out that 31% of marketed drugs were used for different indications than initially defined by the FDA, and the trend has even increased in recent years especially in selected medical areas such as psychiatry, pediatrics or oncology [7,8,9,10,11].

The off-label use of drugs has benefits, but also risks. The benefits are mainly related to the possibility of access to a treatment that has shown its effectiveness against certain morbid conditions (or in certain age groups, gender, etc.) although the indication is not formally approved by regulatory agencies. There are many examples of beneficial off-label use of drugs in clinical practice especially in the oncology field or in the case of rare diseases where the off-label use of drugs or their combinations is often considered the “standard of care”. There are also many cases of drugs whose off-label prescription is so common, which enters the medical practice without patients often even knowing about it (or doctors themselves). Some examples are those related to the use of oral contraceptives for the treatment of menstrual disorders, the use of beta-blockers for the prevention of migraine or the use of phenobarbital for the treatment of an essential tremor, etc. On the other hand, the use of off-label drugs exposes the patient to potential risks mainly related to certain adverse effects that could arise more often or with a worse outcome in a certain population (also referring to the specific pathology) given the fact that the efficacy and safety of these drugs have not been evaluated in that specific category.

The off-label use of vaccines is a topic poorly addressed in the literature. Some examples are the Prevenar conjugated pneumococcal vaccine that was approved with a 3+1 schedule and recommended in Canada with a 2+1 schedule or the use of the Dukoral vaccine, approved against vibrio cholera, also for the prevention of *E. Coli* infections (as claimed by the manufacturer company) [12].

During the COVID-19 outbreak, the lack of effective treatment has led healthcare workers (HCW) to use multiple drugs not specifically tested and approved for the new insidious disease with an unlabeled indication so that an Emergency Use Authorization (EUA) to FDA or to EMA was eventually requested. An EUA was issued for chloroquine and hydroxychloroquine—and then revoked—as well as for other drugs (i.e., Remdesivir) or the monoclonal antibody therapies (i.e., Ely Lilly) [13,14,15].

After the availability of the first COVID-19 vaccines (Comirnaty, Pfizer-BioNTech, and Moderna COVID19 vaccine), the same kind of authorization was also issued by the FDA for these new drugs in order to speed up their introduction on the market and their administration on a large scale. The same formula was also used in Europe by EMA that issued a conditional marketing authorization (CMA) for Comirnaty, Pfizer-BioNTech, Moderna COVID19, and Astrazeneca Vaxzevria vaccine.

Despite the authorization, the off-label use of these vaccines may still be possible especially concerning exogenous factors such as the lack of vaccine doses, the delay in the delivery of planned doses or the pressure from public opinion and political influence also in relation to the evolution of the pandemic.

## 2. Aim and Scope

This paper aims to assess the possible off-label use of COVID-19 vaccines and the ethical and medico-legal implications of this eventuality.

The off-label use of vaccines poses several serious ethical challenges, mostly related to the possible contrast with the WHO concept of rational use of medicines (RUM), which in turn is connected to the principles of risk-benefit balance that in this case is often disengaged from randomized controlled trials (RCTs).

The choice to “deviate” from the recommended path is mostly demanded to the national health system (NHS) or to the single doctor who poses the indication. In the occurrence of any harm to the patient, these subjects could be prosecuted both by the penal and by civil justice for the recognition of financial compensation.

The scope of this paper is to point out the possible consequences of the off-label use of COVID-19 vaccines and possible mitigation and preventive measures to be taken by healthcare workers involved in vaccination procedures.

## 3. Methods

The two different databases (PubMed, Google Scholar, and Web of Science) were questioned using the main keyword “Off-label use” that was crossed with the terms “Drugs”, “Vaccine”, “COVID-19 Vaccine”, and “SARS-CoV-2 Vaccine”.

The selection of the articles was performed by the evaluation of both the title and abstract.

The inclusion criteria were:

- Papers published in English;
- Type of paper: Original article, research article, systematic review, review.

The selected papers that met the inclusion criteria were then reviewed as well as their references. Extensive research on the three COVID-19 vaccines approved in Europe and Italy (Pfizer-BioNTech (Mainz, Germany), Moderna (Cambridge, MA, USA), and AstraZeneca (Cambridge, UK)) was also performed on the websites of national and international Drug Regulatory Authorities (FDA, EMA, AIFA).

## 4. Results and Discussion

The total number of articles collected crossing the chosen keywords was 209 on the Pubmed database, 100 (most relevant articles) on the Google Scholar database, and 131 articles on the Web of Science database. In the review process 36 articles were selected, evaluating the title and abstract. Of these, 19 were eliminated since they were judged as not consistent with the aim of the study. In addition to the remaining 17 articles, other 17 pertinent documents coming from the websites of national and international Drug Regulatory Authorities were also reviewed.

The use of vaccines on a large scale is subject to authorization by various regulatory bodies that approve the drug for a specific indication, with a clear schedule, in an established posology, and through a well-determined administration method. The approval is based on evidence that assesses and supports the quality, safety, and efficacy of such drugs in a certain population. This evidence comes from results of pre-licensure trials in which selected groups were enrolled with strict inclusion and exclusion criteria. The target population of the immunization activity conversely is not included in the group considered for the pre-licensure trial. In this sense, the “real life” utilization may significantly differ from the one recommended by label indications so that the off-label use is indeed realized and sometimes promoted by local NHS recommendations (“off-label public health use”) [12].

Differences will arise whenever a vaccine SmPC/Fact Sheet contains warnings and restrictions against use in certain population groups or according to a precise administration schedule. These limitations mostly derive from the lack of evidence of safety in these groups, for example, relating to difficulties in the enrollment of some population subgroups (e.g., ancient people, people affected by chronic conditions), as well as referring to ethical issues (e.g., pregnant women or women who are breastfeeding).

The NHS may also recommend the use of vaccines for groups not included in the pre-licensure trials based on public health needs/urgencies, coming from more or less structured risk-benefit analysis data performed on post-marketing data or demanding this decision to the single case evaluation performed by the physician.

To date, four vaccines have been authorized in Europe and Italy for the prevention of COVID-19 but only three have been introduced on the market: Pfizer-BioNTech mRNA vaccine (Comirnaty), the Moderna mRNA vaccine, and Vaxzevria vaccine AstraZeneca (viral vector vaccine).

The first vaccine to be authorized was Comirnaty, on 11 December 2020, when the FDA issued the first EUA that allowed the distribution of the drug in the US. Subsequently, on 21 December 2021 EMA granted a CMA for the drug. The same path was also followed shortly after for the Moderna Vaccine while, differently from the two mRNA vaccines, AstraZeneca Vaxzevria has not yet been approved by the FDA but only by EMA. A few days after the EMA authorization, the Italian Drug Authority (AIFA) approved the use of Vaxzevria vaccine, however limiting its use for people aged 18–55 years, for the lack of evidence on vaccine efficacy in persons aged >55 years. Given the new international recommendations, including the opinion of the WHO SAGE group, AIFA expanded the use of Vaxzevria vaccine for subjects aged 56–65 years and after for older people (>65 years).

The main characteristics and indications of the three COVID19 vaccines are summarized in Table 2.

Five hypotheses on the off-label use of COVID-19 vaccines are worth considering, as they appear to most likely have an objective feedback in the short-term. This could happen both in relation to the already evident lack of vaccine doses and to the pressure from public opinion and political influence.

These hypotheses are:

1. Administration to subjects with a personal history of COVID-19;
2. Administration of a single dose of vaccine;
3. Administration in an age group different than recommended;
4. Administration of the second dose of vaccine with an interval different than the one provided in the official schedule;
5. Administration of the second dose with a different vaccine.

The administration of the vaccine to people with a personal history of COVID-19 is a controversial theme especially from an ethical point of view. In the context of limited availability of vaccines, it is clear that if the vaccine is administered to someone who already had the disease, someone else—immunologically naive—will not be immunized or will receive the vaccine later with an undue increase in the risk of contagion [24]. Indeed, if on the one hand, it is certain that the latter subject would benefit from the vaccination, on the other hand, the administration on the former subject although not expressly contraindicated by the “label” could be considered not responding to the logic of clinical rationality (the healed subject could still be covered by natural immunity) or to the concept of rational use of medicines (RUM), advanced by WHO in 1985. In fact, WHO states that the use of medicines is rational when patients receive medications appropriate to their clinical needs, in doses that meet their requirements, for an adequate period of time, and at the lowest cost to them and their community [25].

Referring to natural immunity, Dan et al. highlighted that a substantial immune memory is generated after COVID-19 and that 95% of the 188 subjects included in the study retained immune memory at ~6 months after infection [26]. Similarly, Lumley et al. investigated the incidence of SARS-CoV-2 infection in seropositive and seronegative HCW finding out that the presence of anti-spike or anti-nucleocapsid IgG antibodies was associated with a substantially reduced risk of SARS-CoV-2 re-infection for 6 months after the natural infection [27].

Basically, the latest evidence suggests that a substantial part of healed subjects develop an immunological memory able to drastically reduce the risk of contagion at least up to 6 months after infection. However, this cannot justify the delay of the vaccine in these subjects as stated also by WHO, which clearly indicated that performing tests to detect the presence of SARS-COV-2 antibodies is not recommended for the decision-making on vaccination [28].

Moreover, given the high levels of effectiveness and safety achieved by COVID-19 vaccines, the request from the so-called “frontline workers”, who legitimately require an increased level of protection against an increased risk of contagion, appears absolutely reasonable especially in relation to the wide variability and still poorly understood persistence of natural immunity.

A possible solution has been recently adopted in Italy, proposed by AIFA, and validated by the Scientific Technical Committee of the National Institute of Health. According to the Italian formula, the administration of a single dose of vaccine can be considered in subjects with a previous SARS-Cov-2 infection (symptomatic or asymptomatic) authorizing de facto an “off-label public health use” of COVID-19 vaccines in these cases [29]. According to AIFA, the dose should be administrated to these subjects at least 3 months after the infection and preferably within 6 months consistently with the latest evidence on the persistence of natural immunity. This is not intended to apply to subjects who have immunodeficiency conditions, primitive or secondary to pharmacological treatments, since in these subjects the immunological protection conferred by the infection and the long-time immunogenicity is not foreseeable, thus it is recommended to continue with the proposed vaccination schedule.

Conversely, the administration of a single dose in subjects who have not yet developed the infection or the administration of the second dose following an alternative time schedule (mostly delaying it) is clearly an off-label use of the vaccine. In the context of dose deficiency, this strategy was proposed by many in order to speed up the vaccination process. The rationale of this choice lies in recent studies which have shown the high efficacy of vaccines even with a single dose or with delayed administration of the second dose.

In this regard, we have to clarify that a general principle of vaccinology is that the vaccination cycle should not to be restarted in the case of a prolonged interval between doses (for vaccines requiring multiple doses according to the label).

In a recent analysis, Skowronski et al. concluded that before the second dose, BNT162b2 (Pfizer COVID-19 vaccine) has an efficacy of 92.6% recommending that “with such a highly protective first dose, the benefits derived from a scarce supply of vaccine could be maximized by deferring second doses until all priority group members are offered at least one dose” [30]. Similarly, also the Moderna COVID-19 vaccine efficacy after the first dose was reported to be 92.1% in an FDA briefing document, published on 17 December 2020 [31]. While a single standard dose of the AstraZeneca vaccine was shown to maintain 76% efficacy (95% CI, 59.3–85.9%) against primary symptomatic COVID-19 in the first 3 months after vaccination according to a recent survey, suggesting that a 3-month dose interval might have advantages over a short-dose interval [32].

However, these data on an alternative vaccine schedule (also referring to the length of protection after the first dose) are limited, therefore given the current vaccine shortage the use of a different time schedule should be carefully considered. Indeed, if on the one hand, a larger vaccination coverage could actually provide a short-medium benefit (also for the limitation of wild virus circulation), on the other hand, a strategy based on data disengaged from RCTs can fail in the long-term, transforming the vaccination campaign into an uncontrolled human experimentation with relevant ethical implications [33,34]. Moreover, doubts would arise on the efficacy of the vaccine campaign within the public opinion with the risk, on the one hand, of creating false feelings of safety in patients. On the other hand, in the case of infection in vaccinated subjects, significant damage would occur to the entire vaccination campaign in terms of public confidence with a decrease in voluntary adherence to vaccination.

The administration of the vaccine in an age group other than recommended is another possibility that fully falls in the context of off-label use of the drug according to the shared definitions.

This could happen in a number of cases of vulnerable subjects (i.e., oncohaematological patients), for which the doctor could prescribe the administration of the vaccine even outside the age range recommended by the SmPC/Fact Sheet. This decision would result from a weighted benefit-risk assessment delegated to the opinion of the individual prescriber doctor and therefore disengaged from clinical studies (not available at the moment) especially in subjects under the age of 16 (Comirnaty) or the age of 18 (Moderna, AstraZeneca). The ethical implications of this choice are relevant since they place the doctor in front of a risk in any case: On the one hand, the prescription of a drug that has not been tested in that age group, and on the other hand, the lack of prescription of a drug which is protective against a disease potentially serious or lethal considering the comorbidities of the patient.

Moreover, a particular case is that of AstraZeneca vaccine whose use has no upper age limit for EMA, while it can be administered only up to 65 years for AIFA [22,23]. In the occurrence of other vaccine deficiencies, this could lead to extending its use in Italy also to older people for which at the moment the same problem just reported for those under 16/18 years would occur. However, in this case, a solution could be to integrate within the informed consent also the EMA indications so that, risk-weighting aside, adequate information on the current differences between Drug Regulatory Agencies could be provided to the patient.

The last but not least hypothesis of off-label use is that of the administration of the second dose with a different COVID-19 vaccine. This eventuality may occur in the case of failure to supply the doses already provided, in the event of a mismanagement of the doses already stored or in the exceptional case in which the vaccine product given for the first dose is indeterminable or no longer accessible (this hypothesis has been formulated recently, after the decision of some European Government to temporary suspend AstraZeneca Vaxzevria vaccine). As stated also by the CDC Interim Clinical Guidance, COVID-19 vaccines are not interchangeable and both doses of the series should be completed with the same product since safety and efficacy in a mixed-product series have not been evaluated yet, therefore this eventuality is fully an off-label use [35]. This contingency could be widely prevented through correct programming of the supplies with binding contractual forms, as well as through the correct management of the supplies with prompt registers compilation and in general through a correct managerial administration of the vaccination campaign. In this way, the possibility of using different vaccines in the series would become residual and limited to a very small number of cases for which it would still be advisable to defer the second dose to allow the use of the same product. In the latter case, the same problems related to the single-dose use would arise.

Ethical implications aside, it is clear that such a framework, both in the case of off-label use, is promoted by the NHS (“off-label public health use”) and in this case, the decision is left to the individual doctor prescriber in which medico-legal issues may arise as a result of these choices, especially in the case of any harm to the patient.

Indeed, if the off-label use of the COVID-19 vaccine results in death or personal injury to the patient, the single prescriber doctor could be prosecuted by the penal justice for the related offenses (art. 589–590 of the Italian penal code). This eventuality in Italy has already occurred and has led trade associations to request a “penal shield” for the vaccinator personnel that is currently under consideration by the government.

Referring to civil enforcement, any harm to the patient resulting from the off-label use of COVID-19 vaccines may result in a claim for compensation which, in the light of the current Italian Legislation (Law no. 24/2017), could more likely have an impact on the single healthcare facility finances and therefore on the NHS resources and ultimately on the State coffers. Moreover, considering that COVID-19 vaccines are provided in a National Immunization Program, any damage may be also compensated according to Law no. 210/1992 that recognizes compensation to persons irreversibly damaged by vaccinations, transfusions, and administration of infected blood products.

Referring to vaccines, the law provides compensation if there is a causal link between the administration of a compulsory vaccine and a permanent damage to health following the principle of “social solidarity” not so much aimed at repairing unjust damage, but rather to balance the individual sacrifice when it corresponds to a collective benefit. This may be the case of HCW or other frontline categories who are required to be vaccinated. Again, the compensation costs would be borne by the State, although the economic size of the compensation payments so far provided is unknown, as no data were ever published in Italy on this theme.

Considering the potential economic and social impact of possible vaccine damage in a mass vaccination campaign, it is therefore clear that in order to ensure the safety of patients and the adequate protection for vaccinators, it is necessary to implement effective “barriers” to reduce the risk of harm to the patient so that the proportions of litigation may be at least reduced.

The first barrier is adequate informed consent. According to the Italian Legislation (Law no. 219/2017), informed consent is the prerequisite for the lawfulness of the medical act, since no medical treatment may be initiated or continued prior to the free and informed consent from the person concerned, except in cases expressly required by law. The informed consent has to be complete, up-to-date, and comprehensible to the patient regarding the benefits and risks of the proposed medical treatment and should also include the possible alternatives and consequences of any refusal.

Therefore, in the case of proposed off-label use of COVID-19 vaccines, the information given to the patient must be as complete as possible, including indications from foreign or supranational regulatory agencies and related supporting studies. Particular attention shall be paid to any risks arising from the administration, which must be clearly explained both in relation to a particular category of patients and to the specific vaccination schedule and possible alternatives in the case of force majeure (postponement of the second dose, single-dose administration). In addition, the meaning of the type of authorization granted by regulatory agencies (EUA, CMA) should also be included in the informed consent. Unfortunately, not every data on immediate or long-term adverse effects of vaccines are available at the moment, therefore no informed consent could ever be comprehensive of all possible aspects. Indeed, as demonstrated by recent cases of thrombosis (whose correlation with vaccines has not yet been unequivocally demonstrated), it is necessary to promptly integrate any complications detected in the pharmacovigilance activity in the label and informed consent in order to ensure the full respect for the right to self-determination.

A further “barrier” that could be implemented is the one related to the behavior of vaccinating staff. It is in fact primarily necessary to establish shared paths to be adopted by doctors and health professionals and promote a cultural change in order to minimize the risk for the patient and increase the safety of care.

The tools which can be used are those belonging to Clinical Risk Management, which can be successfully tailored to the new pandemic context and provide a decisive contribution to the vaccination campaign in terms of safety and appropriateness of care. In this sense, in order to protect patients and the medical staff, it could be useful to draw up prior protocols, operating procedures, and therapeutic paths encompassing different circumstances in which the off-label use of COVID vaccines may be required.

The prerequisite for the drafting of such “behavioral normalization” tools is the understanding of the initial conditions and the identification of the objectives to be pursued in terms of clinical excellence and quality of care. Therefore, a clinical audit should be carried out through a structured systematic methodology (also using tools adapted from other contexts as the SWOT analysis) so that the strengths and weaknesses of the system may be highlighted [36,37]. Moreover, to ensure the monitoring of assistance levels in relation to the defined objectives, the strategic direction should introduce adequate performance indicators, and promote the attitude of HCW towards proper pharmacovigilance, reporting of near-miss events, adverse events, and sentinel events related to the off-label administration of COVID vaccines [38].

## 5. Conclusions

In the current condition of lack of COVID-19 vaccine doses and RCTs to certify the effectiveness and safety of the administration in all priority categories, the off-label use of vaccines proposed by many and implemented by several HCW in “real life” is a possibility that cannot be neglected. The administration of the vaccine to subjects with a personal history of COVID-19, using a single dose (when the vaccine is authorized in a two-dose schedule), using a second dose different from the first one, with a time interval other than the one provided in the official schedule or in an age group different than recommended are the most probable off-label uses of COVID-19 vaccine.

As with all drugs used off-label, there is an intrinsic risk that, in the case of damage to the patient, may result in a penal action or a claim for compensation. This occurrence would have a negative impact from the economic and social point of view causing damage to the entire vaccination campaign that could result in a decrease of voluntary adherence by the population. In order to mitigate this risk, the accurate management of vaccination procedures is essential and can be pursued through an adequate informed consent and through the application of tools belonging to Clinical Risk Management that could be successfully tailored to the new pandemic context. This may contribute to the effectiveness of the vaccination campaign increasing public confidence in health authorities.

## Figures and Tables

**Table 1:** Definition of “off-label use” according to FDA, EMA, and AIFA.

Drug Regulatory Agency	Definition
FDA [4,5]	Off-label use is called “unlabeled indication” and is only referred to the physician who prescribes a certain drug to treat a patient for an unlabeled indication.
EMA [2,3]	Use of a medicine for an unapproved indication or in an unapproved age group, dosage or route of administration.
AIFA [1]	Treatment with a medicinal product regularly on the market but for a different indication from the one for which it was authorized, even in the presence of regularly authorized therapeutic alternatives.

**Table 2:** Summary of COVID-19 vaccine characteristics according to FDA, EMA, and AIFA.

Vaccine	Approval	Section	Text from Fact Sheet/SMPC
Pfizer-BioNTech (Comirnaty) mRNA vaccine [16,17,18]	FDA EMA AIFA	Posology and method of administration	Administered intramuscularly after dilution as a cycle of two doses (0.3 mL each) at 21 days from each other.
		Therapeutic indication	Prevention of COVID-19 in individuals aged 16 years or older.
		Pregnancy	The administration should be considered when the potential benefits outweigh any potential risks for the mother and fetus.
Moderna Vaccine mRNA vaccine [19,20,21]	FDA EMA AIFA	Posology and method of administration	One dose (0.5 mL) contains 100 micrograms of messenger RNA (mRNA) (embedded in lipid nanoparticles containing the lipid SM-102), a two-dose course (0.5 mL each). It is recommended that the second dose be administered 28 days after the first.
		Therapeutic indication	Prevention of COVID-19 in adults aged 18 years and over.
		Pregnancy	The administration should be considered when the potential benefits outweigh any potential risks for the mother and fetus.
AstraZeneca Vaxzevria Vaccine Viral vector vaccine [22,23]	EMA AIFA	Posology and method of administration	The COVID-19 Vaccine AstraZeneca vaccination course consists of two separate doses of 0.5 mL each, the second dose should be given 4 to 12 weeks (28 to 84 days) after the first dose.
		Therapeutic indication	Prevention of COVID-19 in adults aged 18 years and over (EMA); In subjects between 18 and 65 years, for which more solid evidence is available (AIFA).
		Pregnancy	The administration should be considered when the potential benefits outweigh any potential risks for the mother and fetus.
